# Clinical experience implanting a miniature externally powered vagus nerve stimulator

**DOI:** 10.1016/j.neurot.2025.e00625

**Published:** 2025-06-26

**Authors:** Joseph D. Epperson, Michael L. Foreman, Richard C. Naftalis, Rita G. Hamilton, Mark B. Powers, Holle L. Gallaway, Gregory dePrisco, Seth A. Hays, Michael P. Kilgard, Robert L. Rennaker, Jane G. Wigginton

**Affiliations:** a Texas Biomedical Device Center, 800 W. Campbell Road, Richardson, TX 75080-3021, USA; b Department of Bioengineering, Erik Jonsson School of Engineering and Computer Science, The University of Texas at Dallas, 860 N Loop Rd, Richardson, TX 75080, USA; c Division of Acute Care Surgery, Baylor University Medical Center, 3500 Gaston Ave, Dallas, TX 75246, USA; d Department of Neurosurgery, Baylor University of Medical Center, 3500 Gaston Ave, Dallas, TX 75246, USA; e Baylor Scott and White Institute for Rehabilitation, 909 N Washington Ave, Dallas, TX 75246, USA; f Baylor Scott and White Research Institute, 3434 Live Oak St Ste 501, Dallas, TX 75204, USA; g Department of Radiology, Baylor University Medical Center, 3500 Gaston Ave, Dallas, TX 75246, USA; h Department of Neuroscience, School of Behavioral and Brain Sciences, The University of Texas at Dallas, GR41, 800 W Campbell Road, Richardson, TX 75080-3021, USA; i XNerve Medical Inc, 500 N. Central Expressway, Suite 500AC, Plano, TX 75074, USA

**Keywords:** Vagus nerve stimulation, Neuromodulation, Stroke, Spinal cord injury, Post-traumatic stress disorder

## Abstract

Vagus nerve stimulation (VNS) is widely used to treat various neurological and psychiatric conditions, including epilepsy and treatment-resistant depression, as well as to enhance motor rehabilitation following stroke. Conventional VNS devices have demonstrated reliability over decades of use, though recent advancements in technology offer new opportunities to further enhance the device. Many emerging indications require only intermittent stimulation, allowing for the development of a miniature externally powered implantable stimulator (MEPS) that is approximately 50 times smaller than conventional devices, is implanted with a single incision, has no battery or leads, and enables paired stimulation. Our observations compiled from three clinical trials with the MEPS device tested the hypotheses that removing the implanted battery and reducing device size would (1) shorten the surgical procedure and (2) maintain or improve safety outcomes. Data were collected from individuals with stroke, spinal cord injury, or post-traumatic stress disorder. Operative time was significantly reduced, averaging 38 ​± ​1 ​min compared to 76 ​± ​3 ​min for conventional VNS, with no significant intraoperative complications and no revision surgeries. The MEPS device successfully delivered 481,995 stimulations during 2205 ​h of therapy. One participant underwent an MRI outside the study, indicating compatibility with standard imaging protocols. Device-related adverse events occurred at a low rate; all were mild and resolved before study end. Overall, the MEPS device demonstrated a favorable safety and performance profile in a select population, with fewer occurrences of certain adverse events, extending the strong safety record of conventional VNS systems.

## Introduction

Vagus nerve stimulation (VNS) has become a widely utilized therapeutic intervention for various neurological and psychiatric conditions, including epilepsy and treatment-resistant depression, and has more recently been applied to support motor rehabilitation after stroke [1]. In recent years, there have been significant advancements in the use of VNS to treat a wide range of conditions including immune system modulation, post-traumatic stress, and motor recovery following spinal cord injuries [[2], [3], [4], [5], [6], [7]]. VNS therapy was first approved by the FDA in 1997 for the treatment of epilepsy, following evidence that demonstrated significant reductions in seizure frequency in individuals with pharmacoresistant epilepsy [8]. Subsequently, in 2005, VNS received approval for the treatment of depression in individuals who did not responded to conventional therapies [9]. In 2021, Paired VNS therapy, which utilizes short bursts of VNS concurrent with rehabilitation, received FDA approval for the treatment of upper extremity motor deficits associated with chronic ischemic stroke [[10], [11], [12]]. Today, VNS continues to be a widely adopted and evolving therapy, demonstrating its versatility and growing relevance in the treatment of numerous complex medical conditions.

First introduced over 25 years ago, the conventional VNS device has demonstrated safety and efficacy for decades in more than 125,000 procedures, though general advancement in technology offer opportunities for improvement [13,14]. Modern developments in device miniaturization, wireless communication, and materials science present the potential to enhance comfort and reduce the need for additional surgical interventions, particularly in cases of lead failure or battery depletion [15,16]. Additionally, improving the compatibility of these devices with routine diagnostic tools such as MRI could streamline clinical care, especially for individuals with neurological injuries who often require regular imaging. These advances aim to address current limitations while maintaining the well-established therapeutic benefits of VNS.

**Fig. 1:**
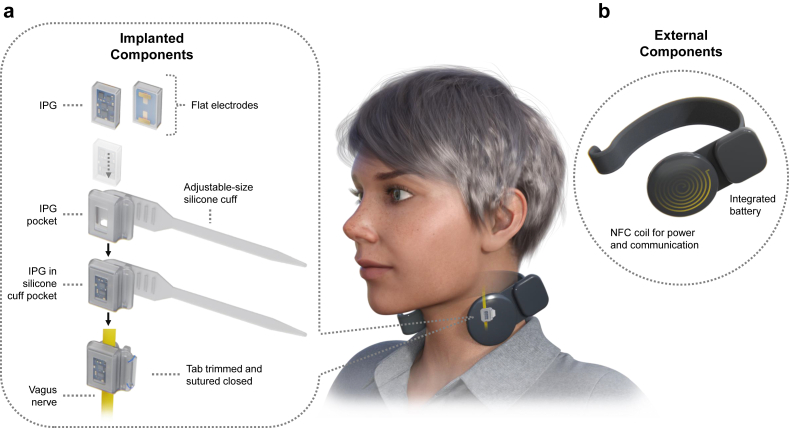
MEPS system description. Overview of the implantable miniature externally powered stimulator (MEPS) system. (Left) The implantable pulse generator (IPG) is encapsulated in biocompatible glass and housed in an adjustable-size silicone cuff. The cuff is secured around the vagus nerve, with the tab trimmed and sutured closed. (Right) The power and communication module (PCM) is displayed with its adjustable plastic neckband in a C shape, which is available in various sizes to fit a variety of neck diameters. The PCM uses inductive coupling to power and communicate with the implanted MEPS, facilitating wireless control and stimulation delivery.

We previously reported the technical development of a miniature externally powered stimulator (MEPS) device, shown here in Fig. 1 [17]. Here, we chronicle our experiences with implanting and utilizing the MEPS device across 49 individuals in three clinical trials. The MEPS device, over 50 times smaller than conventional VNS devices, eliminates the leads and onboard battery typically required, relying instead on wireless power. This drastic size reduction simplifies the surgical procedure, reduces the duration of surgery, and minimizes cosmetic impacts. Furthermore, the device's design eliminates the need for additional procedures to replace the battery and allows compatibility with standard MRI imaging at 1.5T and 3.0T.

Our objective in this exploratory observational study is to evaluate the safety, device reliability, and useability associated with this device. We compiled data from three trials clinical trials using the MEPS device: stroke, spinal cord injury, or post-traumatic stress disorder at a single center. We expected notable differences between the MEPS device and the conventional stimulator, including decreased implant procedure duration, similar or improved adverse event rates, no revision surgeries due to lack of implanted battery or leads, and a high degree of reliability indicated by successful stimulation rates across a high degree of variability in vagus nerve size and depth. Here, we present the experience of 49 individuals, demonstrating an initial assessment of safety, usability and reliability associated with implanting the MEPS device in these populations.

## Materials and Methods

### Study design

This investigation examined the clinical experience of participants in three clinical trials, all of which are early feasibility studies of the MEPS device. Three different indications were examined across the studies: (1) upper limb motor recovery after stroke or (2) spinal cord injury, and (3) treatment resistant post-traumatic stress disorder (PTSD). All procedures were approved by the Institutional Review Board at The University of Texas at Dallas and the IRB at Baylor Scott and White Research Institute in Dallas, Texas. All participants signed a written informed consent before their study participation. Additional study information is available at www.clinicaltrials.gov (NCT04534556, NCT04288245, NCT04064762). All surgical and experimental procedures were performed at Baylor University Medical Center Dallas.

### Components of the system

The MEPS is designed with integrated electrodes and is compact enough to fit into confined anatomical spaces to reduce cosmetic issues and minimize tissue damage during implantation. The MEPS is approximately 50 times smaller by volume than the conventionally available VNS stimulator for motor rehabilitation (MEPS: 13 ​mm ​× ​8 ​mm x 3 ​mm; Vivistim IPG 62 ​mm ​× ​48 ​mm x 12 ​mm [18]). Additionally, the MEPS weighs less than 1 ​% of the conventional IPG (MEPS: ∼0.5 ​g; Vivistim IPG: ∼70.0 ​g, Vivistim Implantable Device Manual, 2021).

The small printed circuit board (PCB) is encapsulated in biocompatible glass (Schott Borofloat 33) using a micro-electro-mechanical systems (MEMS) wafer-level process to ensure consistency, hermeticity, and durability. The MEPS is secured to the nerve by a silicone cuff (NuSil MED-4840), which can adjust to nerves with diameters ranging from 2 ​mm to 6 ​mm, aiming to facilitate proper current flow inside the cuff and through the vagus nerve to minimize off target effects. The MEPS devices store the stimulation parameters, permitting blinding during clinical trials and avoiding errors when switching external controllers. The MEPS delivers charge-balanced, biphasic current pulses, with programmable amplitudes, pulse widths, frequencies, and train durations. This design permits tailored therapeutic interventions, used here to deliver blinded sham stimulation or active stimulation with varied stimulation intensities (up to 1.2 ​mA). Standard parameters for Targeted Plasticity Therapy (TPT) were used in the three clinical studies associated with this research (Current: 0.8 ​mA; pulse width: 100 μsec; frequency: 30 ​Hz; burst duration: 0.5 ​s).

An externally worn relay module, known as the power and communication module (PCM) is placed in a neckband and positioned on the skin above the IPG during use. The PCM, powered by a rechargeable battery, provides wireless power and communication to the IPG through inductive coupling. The PCM also wirelessly communicates via Bluetooth with a programming application on an Android tablet or phone, allowing clinicians to set stimulation parameters and trigger stimulation as needed. The MEPS device and peripherals have been fully described in previous work [17].

### Surgical procedure

All 49 eligible enrolled participants were implanted with the MEPS device. Implantation was performed by a general surgeon with no prior VNS experience, assisted by a neurosurgeon with experience in conventional VNS systems. After surgical preparation and induction of general anesthesia, the patient was positioned with the head extended and turned to their right. A transverse incision was made at the level of the cricoid over the anterior border of the sternocleidomastoid muscle, which was reflected laterally to expose the carotid sheath. The internal jugular vein was reflected laterally to visualize the vagus nerve, blunt-teeth Weitlander retractors were used to maintain exposure, and a 3 ​cm segment of the nerve was dissected free to allow placement of the device (Supplementary Fig. 2). Before placing the device, the depth and diameter of the vagus nerve for each participant was recorded. The long tab of the silicone cuff was threaded around the nerve and passed through the strap on the cuff, similar to a zip tie. The long tab was pulled through the strap until the MEPS electrodes were in contact with the nerve. The cuff around the device was closed with two 4-0 permanent sutures but was not attached to the muscle or muscle fascia. The long tab of the silicone cuff was trimmed and removed, then the MEPS body was positioned anterior to the nerve and parallel to the skin to facilitate alignment with the external components during stimulation. After removing retractors, wireless communication and power functions of the MEPS were verified. Following confirmation of device functionality, 0.25 ​% bupivacaine with epinephrine was injected into the incision, and the platysma and skin were closed with absorbable sutures. A second communication verification was performed before the sterile field was broken, followed by an application of cyanoacrylate tissue adhesive.

Routine post-anesthetic care was provided with discharge once fully awake. Pain management was effectively controlled with over-the-counter medications, such as acetaminophen or non-steroidal anti-inflammatory drugs (NSAIDs) and the use of an ice pack. Approximately two weeks later, participants returned to the clinic for a follow-up visit. During this appointment, the surgical site was evaluated for healing progress and any complications. In addition, postoperative assessments were conducted to reassess vocal quality and swallowing function.

### Transient vocal response to stimulation

Participants were positioned in a quiet environment to test their vocal response to VNS. Each participant was subjected to pseudo-randomized VNS intensities across a series of runs, with a control (no stimulation) condition as the first run. The current intensities ranged from 200 ​μA to 1200 ​μA, with each intensity applied for five repetitions. Participants were instructed to vocalize a sustained vowel sound (“a” as in “father”) while VNS was delivered approximately one to two ​seconds after vocalization began. If no vocal response was observed at lower intensities, subsequent higher intensity runs were administered. Vocal responses were evaluated by both the participant's perception and the examiner's auditory observation. After each stimulation, participants were asked if they “felt” the stimulation, and responses were recorded as “Yes,” “No,” or “Kind of.” The examiner scored the vocal response on a scale from 0 to 5, where 0 indicated no detectable change in vocalization and 5 represented an obvious, easily detectable vocal interruption. Intermediate values from 1 to 4 were assigned based on the degree of perceived vocal disruption, with a score of 2 specifically indicating minimal but noticeable disruption.

### Data collection

Personnel were present during all implantation surgeries to gather detailed procedural data, including surgery segment times, device tightness, and implant depth. Following implantation, study participants had regular follow-up interactions with personnel to ensure continued monitoring and data collection. These interactions included routine check-ups, assessments of device functionality, and evaluations of progress and outcomes.

### Data analysis

Data analysis was conducted using custom Python scripts developed for this study and Microsoft Excel for data management and initial statistical computations. Comparative data from previous studies on conventional VNS devices were directly extracted from published sources and have not been further subcategorized or processed in any way. The primary comparative data was collected from Liu et al., which studied the implant experience of 108 patients with a conventional VNS stimulation, including typical adverse event rates, surgical duration, and other relevant data. These data were used as benchmarks for evaluating the performance of the device in the current study.

### Statistics

Data are reported as mean ​± ​standard error of the mean (SEM). Where appropriate, standard parametric statistical tests (unpaired t-tests) were used to make comparisons. Statistical tests for each comparison are noted in the text. A Kruskal-Wallis test was used to determine if surgical times across indications varied significantly, and unpaired two-tailed t-tests were used to determine differences in the overall surgery duration. The threshold for statistical significance was set at p ​< ​0.05. Error bars in figures represent SD.

## Results

### Surgical outcomes and recovery

**Fig. 2:**
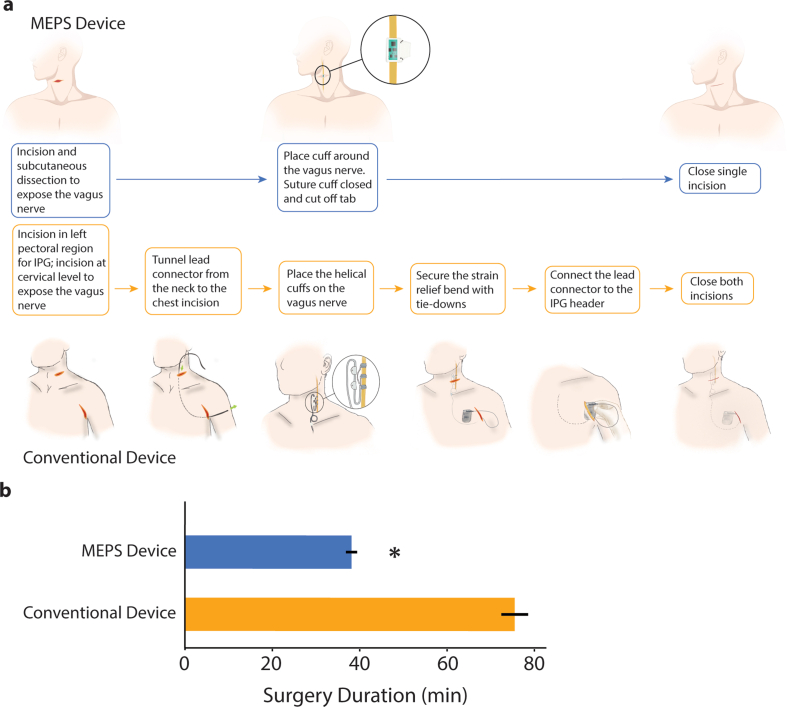
Comparison of implantation procedures and surgery duration for the MEPS and conventional device. (A) Illustration of the steps involved in implanting the MEPS and conventional devices. Implantation of the MEPS device involves a single incision to expose the vagus nerve, placing the isolated nerve in a silicone cuff containing the MEPS device, and closing the skin incision. Implantation of the conventional device requires additional steps to account for placing and connecting the IPG with the leads. (B) Owing to the reduced complexity, the implantation of the MEPS device was significantly shorter in duration than the conventional device. Conventional device procedure adapted from Liu et al., 2022.

The surgical implantation process for VNS devices differs significantly between the conventional system and the MEPS device, both in terms of procedure complexity and operative time. The MEPS procedure involves a single incision to expose and secure the vagus nerve cuff, eliminating the need for lead tunneling and additional chest incisions. In contrast, the conventional system requires both a neck and chest incision, along with a more involved process of tunneling the lead connector and securing it to the pulse generator. These differences lead to notable differences in the time required for each stage of the procedure. The operative time for the MEPS device was shorter than for the conventional device, with a mean duration of 38.1 ​± ​1.3 ​min, compared to 75.5 ​± ​3.1 ​min for the conventional device used for VNS after stroke [18,19], and 94 ​min for a similar VNS device used to treat epilepsy (unpaired *t*-test, p ​= ​2.4 ​× ​10ˆ-21, Fig. 2). Mean approach time was 20.4 ​± ​1.2 ​min, and the mean cuff placement time was 7.6 ​± ​0.9 ​min. Surgical durations for each indication were not significantly different from each other (PTSD: 37.0 ​± ​2.2 ​min; SCI: 37.8 ​± ​2.1 ​min; Stroke: 39.2 ​± ​2.4 ​min, Kruskal-Wallis H statistic: 0.3469, p ​= ​0.8408). The results demonstrate a shorter operative time for the MEPS device compared to conventional devices, with consistent durations observed across different groups.

Every participant who entered surgery received the implant and recovered without long term surgical complications. Ninety-six percent (47/49) of participants were discharged on the same day as the procedure with instructions for postoperative care at home. The remaining two individuals were discharged the following day. Pain management was controlled with over-the-counter medications, such as acetaminophen or NSAIDs. No additional analgesic needs were reported. Approximately two weeks later (16 ​± ​1 days), participants returned to the clinic for a post-op visit, where the surgical site was evaluated for healing progress and any complications. No significant surgical site issues were observed in any of the participants.

Recovery after MEPS device implantation was efficient, enabling most participants to begin therapy soon after their post-op visit. The typical interval between the post-op visit and the first therapy session was 8 ​± ​1 days, with the exception of two individuals who experienced communication challenges between the PCM and MEPS due to neck size. These metrics are comparable to the time reported for the recovery after implantation of the conventional VNS device [18]. Differences between device implantation and start of therapy may be due to scheduling rather than recovery challenges. Thus, participants' rapid recovery enabled timely commencement of therapy, with no major delays attributable to the surgical procedure itself.

### Safety

The safety profile of the MEPS device was evaluated and compared with conventional VNS devices. The analysis included intraoperative and postoperative complications and device performance.

During the implantation of the device, no significant perioperative complications were reported, with the exception of a single case of pneumothorax that occurred as the individual began coughing while coming out of anesthesia. Postoperative monitoring revealed a low rate of adverse events (AE) related to surgery (Table 1). AEs reported as at least possibly related to surgery were unremarkable. Pain at the incision site was the most common AE and occurred in 12 ​% of cases, but all pain-related AEs were resolved prior to study completion. Voice alteration is a known consequence of conventional VNS device implantation but did not occur with the MEPS device (Table 1). Additionally, participants who received the miniature device did not experience other previously reported symptoms associated with conventional device implant, such as edema, fluid at implant, significant bruising, or tremor (Table 1). While we cannot rule out that the lower AE occurrence was due to the reduced sample size, it is plausible that the reduced surgical time and complexity and small device size gave rise to the improved AE rate.

**Table 1:** Comparison of incidence and severity of all surgical adverse events. Subjects are counted once per event at the worse severity. Totals for the number of subjects at a higher level may not equal the sum of the events reported below, as a single subject may report more than one adverse event within the higher-level category. Adverse events in bold italics were experienced by Vivistim users but not experienced in the MEPS study. Skin Irritation, in italics, was not observed in participants with the Vivistim device. Vivistim data derived from Liu et al., 2022.

49 surgeries	MEPS Device		Adverse Event	108 surgeries	Vivistim	
Mild	Moderate	Severe		Mild	Moderate	Severe
11 (22 ​%)	1 (2 ​%)	1 (2 ​%)	Surgical and medical procedures	28 (25.9 ​%)	8 (7.4 ​%)	0 (0 ​%)
6 (12 ​%)	0 (0 ​%)	0 (0 ​%)	Pain	19 (17.6 ​%)	5 (4.6 ​%)	0 (0 ​%)
3 (6 ​%)	1 (2 ​%)	0 (0 ​%)	Hoarseness	4 (3.7 ​%)	0 (0 ​%)	0 (0 ​%)
2 (4 ​%)	0 (0 ​%)	0 (0 ​%)	Paresthesia	1 (0.9 ​%)	0 (0 ​%)	0 (0 ​%)
2 (4 ​%)	0 (0 ​%)	0 (0 ​%)	*Skin Irritation*	0 (0 ​%)	0 (0 ​%)	0 (0 ​%)
1 (2 ​%)	0 (0 ​%)	0 (0 ​%)	Coughing	1 (0.9 ​%)	0 (0 ​%)	0 (0 ​%)
1 (2 ​%)	0 (0 ​%)	0 (0 ​%)	Dysphagia	1 (0.9 ​%)	0 (0 ​%)	0 (0 ​%)
1 (2 ​%)	0 (0 ​%)	0 (0 ​%)	Pharyngitis	1 (0.9 ​%)	0 (0 ​%)	0 (0 ​%)
1 (2 ​%)	0 (0 ​%)	0 (0 ​%)	Nausea	1 (0.9 ​%)	0 (0 ​%)	0 (0 ​%)
1 (2 ​%)	0 (0 ​%)	0 (0 ​%)	Keloid scar	1 (0.9 ​%)	0 (0 ​%)	0 (0 ​%)
1 (2 ​%)	0 (0 ​%)	0 (0 ​%)	Swelling	0 (0 ​%)	1 (0.9 ​%)	0 (0 ​%)
*0 (0 ​%)*	*0 (0 ​%)*	*0 (0 ​%)*	***Voice alteration***	2 (1.9 ​%)	2 (1.9 ​%)	0 (0 ​%)
*0 (0 ​%)*	*0 (0 ​%)*	*0 (0 ​%)*	***Edema, surgical site***	2 (1.9 ​%)	0 (0 ​%)	0 (0 ​%)
*0 (0 ​%)*	*0 (0 ​%)*	*0 (0 ​%)*	***Fluid at implant***	2 (1.9 ​%)	0 (0 ​%)	0 (0 ​%)
*0 (0 ​%)*	*0 (0 ​%)*	*0 (0 ​%)*	***Antibiotics***	1 (0.9 ​%)	0 (0 ​%)	0 (0 ​%)
*0 (0 ​%)*	*0 (0 ​%)*	*0 (0 ​%)*	***Bruise***	1 (0.9 ​%)	0 (0 ​%)	0 (0 ​%)
*0 (0 ​%)*	*0 (0 ​%)*	*0 (0 ​%)*	***Edema***	0 (0 ​%)	1 (0.9 ​%)	0 (0 ​%)
0 (0 ​%)	0 (0 ​%)	1 (2 ​%)	Surgical complication	1 (0.9 ​%)	0 (0 ​%)	0 (0 ​%)
*0 (0 ​%)*	*0 (0 ​%)*	*0 (0 ​%)*	***Tremor***	1 (0.9 ​%)	0 (0 ​%)	0 (0 ​%)

Notably, no adverse events (AEs) were reported in relation to the placement of the PCM on the surgical site, nor were there any issues associated with the long-term use of the PCM and neckband. This outcome suggests that the MEPS device is not only effective but also minimizes the potential for complications typically associated with implantable devices, particularly in areas of prolonged contact with the skin.

Throughout the study, participants demonstrated high levels of device tolerability. Ninety-five percent of participants reported satisfaction with the therapy they received, indicating positive user experience. The PCM was worn for up to 2 ​h per day during therapy sessions, with each participant completing over 150 ​h of therapy without reporting any irritation or discomfort. This high level of satisfaction and comfort underscores the MEPS device's ease of use in long-term therapy applications.

A comparison of adverse event rates between the MEPS device and conventional VNS devices is presented in Table 2, offering data on the safety profiles of both devices in relation to established technologies. The data indicate that the MEPS device's safety profile is at least comparable to, and in some instances potentially better than, conventional devices.

**Table 2:** Adverse event rates are comparable or improved over conventional device. All current study AEs are reported from categories Definitely, Probably, or Possibly device-related. Vivistim data derived from Liu et al., 2022.

Adverse Event	Vivistim	MEPS
Pain due to implant	22.6 ​%	14 ​%
Voice alteration (Hoarseness)	7.5 ​%	8 ​%
Coughing	4.6 ​%	6 ​%
Paresthesia	3.8 ​%	4 ​%
Pharyngitis	3.8 ​%	4 ​%
Nausea	3.8 ​%	2 ​%
Dyspnea	1.9 ​%	0 ​%
Dysphagia	1.9 ​%	2 ​%
Dyspepsia	1.9 ​%	0 ​%
Laryngismus	1.8 ​%	2 ​%

### Device performance

Across three separate studies, device performance and participant experiences with the MEPS system were observed to evaluate its functionality during rehabilitation. All devices passed initial communication checks before the completion of each surgical procedure, indicating readiness for therapy. No revision surgeries were required during follow-up, and all participants received stimulation during therapy as intended, suggesting that the system functioned reliably across the group.

**Fig. 3:**
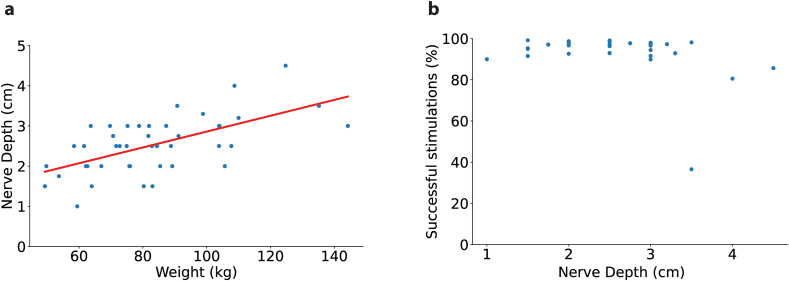
The device communicated successfully for a large range of nerve depths and neck sizes. (Left) The MEPS device (designed for implant depths 4 ​cm or less) demonstrated high efficacy across a wide range of nerve depths, with successful stimulation rates generally above 80 ​%. (Right) Scatter plot illustrating the correlation between nerve depth (cm) and participant weight (kg). The red line represents the linear regression, indicating that nerve depth increases with participant weight (R^2^ ​= ​0.371, p ​= ​1.482 ​× ​10ˆ-5). The findings highlight that while the MEPS device performs well across various nerve depths, participant weight and, consequently, neck size may influence implantation depth and device performance.

The MEPS system appeared to operate consistently across participants with varying physical characteristics, including body weights ranging from 47 to 145 ​kg and vagus nerve depths from 1.0 to 4.5 ​cm (mean: 2.5 ​± ​0.1 ​cm, Fig. 3). Vagus nerve diameters ranged from 2.0 to 3.5 ​mm (mean: 2.8 ​± ​0.1 ​mm), which were within the expected range for the device's operation (Table 3). These variations in anatomy and implant depth did not seem to impact the system's ability to deliver therapy, although further data would be needed to assess performance across a broader population.

**Table 3:** Vagus nerve measurements of study participants. Vagus nerve metrics were obtained through direct measurements during surgery. The MEPS system appeared to operate well across the anthropometric range. Values are reported as mean ​± ​SEM where applicable.

	Diameter (mm)	Depth (cm)
Mean ​± ​SEM	2.8 ​± ​0.1	2.5 ​± ​0.1
Range	2.0–3.5	1.0–4.5

In total, 481,995 stimulations were delivered over 2205 ​h of therapy. One participant, a person from the stroke study, experienced intermittent communication issues between the external PCM and the implanted MEPS device while seated in a specific rehabilitation posture, particularly when leaning forward. Power and communication were restored when the individuals reclined and extended their neck, suggesting that this may be a posture-dependent communication issue. This was the only instance like this observed among participants. Benchtop testing suggested that communication drops were primarily related to device alignment issues. Specifically, misalignment in roll, pitch, and yaw of the implant in relation to the PCM appeared to narrow the effective range for communication and power transfer (Supplementary Fig. 1). These tests simulated various neck movements and positions to better understand potential alignment-related challenges.

Stimulation triggering was performed either manually via a smartphone application during exposure therapy or automatically through an algorithm during motor rehabilitation [20]. The PCM's 550 ​mAh battery appeared sufficient to provide power for up to 2 ​h of therapy per day. Bluetooth Low Energy (BLE) and Near Field Communication (NFC) protocols enabled stable communication, with no interference observed, even when multiple devices were used simultaneously in the same environment.

### Diagnostic imaging after device implantation

**Fig. 4:**
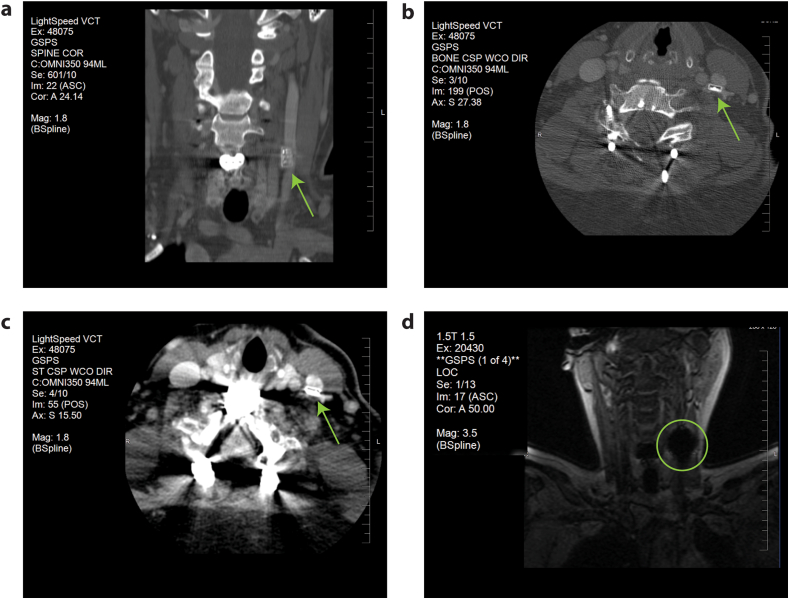
MEPS device is visible in CT imaging and occluded in MRI. The MEPS device is visible in three CT images (A, B, C), with key scan parameters labeled for each. The CT images, using a slice thickness ranging from 2.4 to 5.15 ​mm, provide clear anatomical localization of the device (indicated by green arrows). Magnification (1.8×) and contrast agent (Omnipaque 350, 94 ​mL) enhance visibility in both bone and soft tissue views. The MRI image (D) shows the expected artifact (marked with green circle) caused by the device, which occludes its visibility. However, the size of the artifact does not interfere with the usefulness of 1.5T MRI imaging for routine diagnostic purposes, allowing evaluation of surrounding structures. This combination of imaging modalities demonstrates the device's compatibility with both CT and MRI for routine post-operative care.

As part of routine medical care, a subset of participants in these studies underwent diagnostic imaging procedures unrelated to the primary research protocols. The MEPS device was imaged using computed tomography (CT) and magnetic resonance imaging (MRI), demonstrating compatibility with routine diagnostic workflows. We are aware of three participants who received routine imaging during these studies. Devices remained functional after imaging with no perturbation of performance. In the CT scans from one participant (Fig. 4), the device was clearly visible in both coronal and axial views. The coronal CT image, acquired with a slice thickness of 2.4 ​mm, provided detailed visualization of the device's vertical placement relative to the cervical spine, while axial views with slice thicknesses of 2.97 ​mm and 5.15 ​mm allowed for cross-sectional imaging of its position within the surrounding anatomical structures. The use of a contrast agent (Omnipaque 350, 94 ​mL) enhanced the differentiation of the device from surrounding soft tissues.

In the MRI scan (1.5T, Fig. 4), an expected signal artifact was observed, preventing direct visualization of the MEPS device. This artifact was anticipated due to the device's metallic components and conductive materials, which are known to disrupt MRI signals based on prior testing. Despite the presence of the artifact, the surrounding anatomical structures were still clearly visible, suggesting that standard MRI assessments for routine medical care can proceed without complication.

### Confirmation of vagus nerve activation

**Fig. 5:**
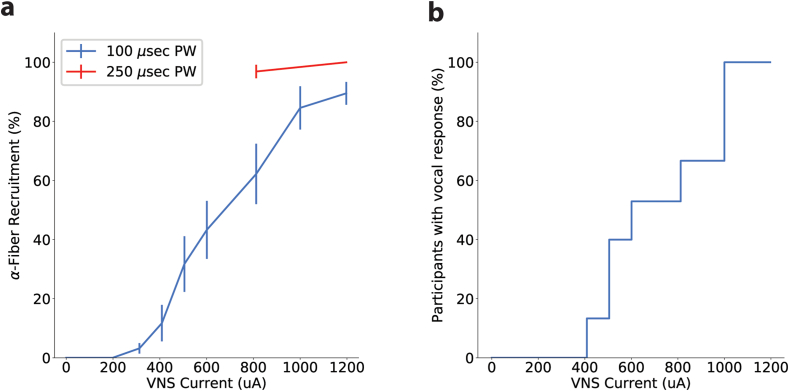
Increasing VNS intensity and pulse width (PW) enhances α-fiber activation. VNS intensity was varied from 0 to 1200 ​μA to investigate α-fiber recruitment by measuring vocal response at each intensity level as a proxy for vagal nerve activation. (A) The plot demonstrates a dose-dependent increase in α-fiber recruitment with higher VNS intensities, where larger currents progressively activate more α-fibers, as indicated by corresponding vocal responses. As expected, increasing the pulse width (PW) from 100 to 250 ​μs ​at the same current resulted in greater α-fiber recruitment due to the higher charge per pulse, which enhanced nerve activation. The y-axis represents the percentage of the maximal vocal response observed, normalized to the highest response across all trials. (B) The plot shows that all participants experienced a vocal response of at least moderate intensity (score ≥2) when the stimulation current exceeded 800 ​μA.

The recurrent laryngeal nerve (RLN) branches from the vagus nerve and provides motor innervation to the intrinsic muscles of the larynx, allowing for vocal cord movement. This nerve contains α-fibers, which are fast-conducting, myelinated fibers capable of eliciting distinct vocal responses when sufficiently stimulated. When activated, these α-fibers produce a transient vocal interruption during the sustained production of vowel sound. The vocal interruptions, as measured by the examiner, were observed at varying intensities across participants, with the highest VNS intensity of 1200 ​μA producing consistently elevated responses. Low intensities (e.g., 200 ​μA) often produced no vocal disruption, as reflected by lower scores (0–2) across trials. As shown in Fig. 5A, there was a dose-dependent increase in the percentage of maximal vocal response with higher VNS intensities, suggesting progressive recruitment of α-fibers at increasing current levels. Additionally, increasing the pulse width from 100 ​μsec, the standard setting in Targeted Plasticity Therapy (TPT), to 250 ​μs ​at the same intensity led to a stronger vocal response (Fig. 5A), consistent with enhanced α-fiber activation due to greater charge delivery.

This recruitment was observed across all participants, with vocal response achieved in 100 ​% of cases, underscoring the reliability of this approach as an external validation of vagus nerve activation (Fig. 5B). Importantly, these results indicate that the MEPS device can reliably stimulate the vagus nerve at intensities sufficient to recruit α-fibers, providing confidence in its functionality without necessitating more complex biosignal measurements. This approach leverages the physiological connection between vagus nerve stimulation and vocal motor response, serving as an independent measure of activation of the cervical vagus nerve and confirming that the MEPS operates as designed.

While VNS produced a detectable change in the quality of a sustained (∼5 ​s) vowel sound, the 0.5 ​s stimulation events did not interfere with vocal production or intelligibility. Most participants and therapists were unable to detect the onset of VNS events during speaking.

### Device retention and follow-up

All implanted devices were successfully activated, and no revision surgeries were required. Follow-up assessments at the end of the study indicated that all participants who completed therapy had fully functional devices. One participant who received a MEPS device did not return for their postoperative examination and was lost to follow-up. At the point of publication, this report reflects over 110 patient years of safety data.

## Discussion

This study presents initial findings from three clinical trials involving the MEPS device for vagus nerve stimulation (VNS). The MEPS device significantly reduced surgical time compared to conventional VNS devices, with a mean duration of 38.1 ​min versus 75.5 ​min. No major device-related complications were reported during or after implantation, and the device successfully delivered 481,995 stimulations across 2205 ​h of therapy. The MEPS device functioned reliably with no revision surgeries required, demonstrating effective stimulation in a range of participant sizes and implant depths.

This study presents several novel contributions to the field of VNS. Firstly, we have successfully implanted VNS devices in individuals with treatment resistant post-traumatic stress disorder (PTSD) and cervical spinal cord injury, expanding the potential therapeutic applications of VNS beyond its traditional use in epilepsy, depression, and stroke. Given the distinct etiology and pathophysiology of spinal cord injury and PTSD—conditions that affect motor function and mental health through very different mechanisms— it was conceivable that these conditions would have different risk profiles. However, our results show that the MEPS device can be safely and effectively implanted in these populations, with surgical outcomes that closely resemble those observed in stroke patients. Similar outcomes across diverse conditions underscore the potential of VNS as a versatile treatment modality, expanding its reach into new therapeutic areas. These results are particularly encouraging for individuals with spinal cord injury or PTSD, where existing treatment options remain limited, and further support the need for continued investigation into the broader clinical applications of VNS.

Secondly, our findings demonstrate that the MEPS device can be implanted in approximately half the time required for the larger, conventional device, which necessitates two incisions. The reduced operative time reflects the smaller size and direct placement on the nerve, demonstrating a reduction in surgical burden. This streamlined procedure may facilitate quicker patient turnover and reduced use of surgical resources. These advancements provide a basis for further research to evaluate the broader applicability and long-term outcomes of VNS therapy using this new device.

Historically, devices such as the VNS Therapy System (Cyberonics, Inc.) have been the standard for vagus nerve stimulation (VNS), offering significant therapeutic benefits but also presenting limitations. These systems involve surgical implantation of a pulse generator beneath the skin of the chest, with leads tunneled to the left vagus nerve at the cervical level. Although effective, clinical studies have reported issues such as lead breakage and battery depletion, both of which can necessitate additional surgical interventions to replace components or address malfunctions [15,16]. The most recent iteration of this design is an FDA-approved stimulator (Vivistim, MicroTransponder Inc.) that uses VNS to improve upper limb motor function in patients with chronic stroke.

In contrast to these semi-continuous VNS systems, which deliver ongoing stimulation throughout the day, emerging applications in rehabilitation rely on short, targeted bursts of stimulation that are delivered only during specific therapeutic activities. This selective approach, referred to as Targeted Plasticity Therapy (TPT), delivers VNS to coincide precisely with rehabilitation exercises, reducing the overall demand for power [1]. This has a direct impact on device design, enabling the removal of the large, implanted batteries required by traditional systems.

Our MEPS system was purpose-built for paired stimulation during rehabilitative exercises, leveraging the reduced power requirements of TPT. Without the need for a bulky battery, the MEPS device is miniaturized, designed to fit seamlessly into confined anatomical spaces, minimizing the risk of cosmetic concerns or tissue damage. Its integrated electrodes and battery-free design ensure that the device does not interfere with the individual's daily life while allowing for routine medical care, including imaging. Previous benchtop testing has confirmed its operational performance, reliability, biocompatibility, and efficacy in nerve recruitment [17].

All adverse events (AEs) and serious adverse events (SAEs) observed were anticipated, and their rates were comparable or lower than those reported for conventional VNS devices [14]. This outcome was expected because our study utilized low dose (0.8ma), 0.5 ​s long bursts of VNS to drive brain plasticity. In contrast, conventional VNS treatments often involve 30-s-long pulse trains as high as 3.5 ​mA [21]. Consequently, the reduced intensity and intermittent nature of the stimulation in our studies likely contributed to the lower incidence of commonly reported adverse events related to stimulation. In addition to these indications of safety, we also showed that standard MRI can be safely performed without the need for device removal.

The MEPS device was designed to accommodate varying neck diameters and anatomical differences, as demonstrated by the successful stimulation across a wide range of implant depths and vagus nerve diameters reported in this study. While neck diameter was not directly recorded, the large range in participant weight (47 ​kg–145 ​kg), along with consistent communication and stimulation success, suggests that the system can function effectively across a broad spectrum of anthropometric measurements. Consistent efficacy was seen across a large range of weight and implant depth, but we empirically observed that, at times, participants with deep implants or large neck diameters presented communication challenges. Future trials should consider a neck-diameter exclusion criterion, barring any updates to the technology that improves the range. It should be noted that due to the implantable controller in the conventional design, it is not constrained by neck diameter and could pose as a viable alternative for patients who would not experience consistent wireless communication with the MEPS device.

The majority of available systems for implanted vagus nerve stimulation use helical stimulation electrodes. The highly flexible nature of this design is an advantage; however, the disadvantages of helical designs include that they are prone to breakage, challenging to implant, and associated with scar tissue formation that can influence current spread [19,[22], [23], [24]]. The MEPS cuff used in this study features a soft silicone body that encircles the nerve, distinguishing it from earlier helical-coil designs. In our preclinical research, we observed the formation of a transparent 300 ​μm thick connective tissue around the exterior of the cuff. This encapsulation facilitates the flow of current through the nerve while minimizing the impact on the surrounding tissue. Modeling and animal studies were used to confirm that the design activates the entire nerve even though the contacts are located on only one side [25]. Importantly, computational and in vivo studies demonstrated similar fiber recruitment thresholds for circumferential electrode and non-circumferential cuff designs [25,26]. These earlier studies combined with the results in humans reported here demonstrate that flat electrodes represent a viable design for nerve stimulation.

Our study, while demonstrating promising results for the MEPS device, has several limitations that warrant discussion. Firstly, the study was conducted on a specific population with strict inclusion and exclusion criteria. This selected group may not represent the broader population of patients who could benefit from VNS therapy, potentially limiting the generalizability of our findings. Additionally, the participants were observationally compared across separate studies rather than directly compared within a single randomized trial, which may introduce confounding factors and limit the strength of comparative conclusions.This study was conducted at a single center, which may introduce site-specific biases and restrict the external validity of the results. Conducting multi-center studies would help confirm these findings and ensure broader applicability. The MEPS device is designed for a maximum nerve depth of 4 ​cm, and this limitation may affect individuals with larger neck circumferences or higher body mass index (BMI). The possibility of initial edema at the surgical site could also impact device performance and patient comfort in the immediate postoperative period. Although we followed participants for two years, additional long-term data on device performance, durability, and patient outcomes are necessary to fully understand the benefits and potential drawbacks of the MEPS device. While the incidence of adverse events was low, continued surveillance is required to identify any long-term complications or issues that may arise with extended use of the device. Technological limitations were noted, including instances of communication challenges between the PCM and the MEPS, particularly in certain postures. Further refinement of the device's communication protocols and hardware may be needed to ensure consistent performance.

The successful implementation of the MEPS device highlights the potential for further advancements in neuromodulation technology. Future research could explore the long-term efficacy and safety of the device, comparing its performance in larger, more diverse populations. Additionally, investigating the device's impact on other neurological conditions and its compatibility with other therapeutic modalities could provide valuable insights. Continued innovation in device design and functionality, coupled with rigorous clinical evaluation, will be crucial in maximizing the therapeutic benefits of vagus nerve stimulation.

## Author contributions

JDE, MLF, RCN, RGH, MBP, HLG, SAH, MPK, RLR, and JGW participated in the design and conception of this study. Data collection was performed by JDE, MLF, RCN, RGH, HLG, RLR, and JGW. Data analysis and interpretation was performed by JDE, MPK, and JGW. Radiologic interpretation was performed by GD. JDE wrote and JGW, SAH and MPK each edited the manuscript. All authors read and approved of the final manuscript.

## Data availability

Due to the sensitive nature of the patient information involved in this study, the primary dataset cannot be publicly shared. However, limited anonymized or aggregated data, such as stimulation metrics, may be made available upon reasonable request to the corresponding author.

## Funding sources

This work was sponsored by the National Institutes of Health UG3/UH3 NS109497 (SAH and RLR), the Communities Foundation of Texas, Wings for Life, and through the Accelerated Translational Program and the Defense Advanced Research Projects Agency (DARPA) Biological Technologies Office (BTO) TNT program under the auspices of Drs. Tristan McClure-Begley, Matt Pava, and Joeanna Arthur through the Naval Information Warfare Center Pacific, Pacific Grant/Contract No. N66001-17-2-4011 and the ElectRx program under the auspices of Drs. Doug Weber, Eric Van Gieson, and Joeanna Arthur through the Space and Naval Warfare Systems Center, Pacific Grant/Contract No. N66001-15-2-4057.

## Declaration of competing interest

The authors declare the following financial interests/personal relationships which may be considered as potential competing interests:Seth Hays, Robert Rennaker reports financial support was provided by National Institutes of Health. Michael Kilgard reports financial support was provided by Communities Foundation of Texas. Michael Kilgard reports financial support was provided by Wings for Life Spinal Cord Research Foundation. Michael Kilgard, Seth Hays, Robert Rennaker reports financial support was provided by Defense Advanced Research Projects Agency Biological Technologies Office. Robert Rennaker reports a relationship with XNerve Medical that includes: board membership and equity or stocks. Joseph Epperson reports a relationship with XNerve Medical that includes: consulting or advisory. Michael Kilgard reports a relationship with MicroTransponder, Inc. that includes: equity or stocks. If there are other authors, they declare that they have no known competing financial interests or personal relationships that could have appeared to influence the work reported in this paper.
