# Supplementary material for: Clinical experience implanting a miniature externally powered vagus nerve stimulator
Source: Neurotherapeutics. 2025 Jun 26;22(5):e00625. doi: 10.1016/j.neurot.2025.e00625 (PMC12491804; doi:10.1016/j.neurot.2025.e00625)
Supplement: Multimedia component 1 [file mmc1.docx]

**Supplementary Data**


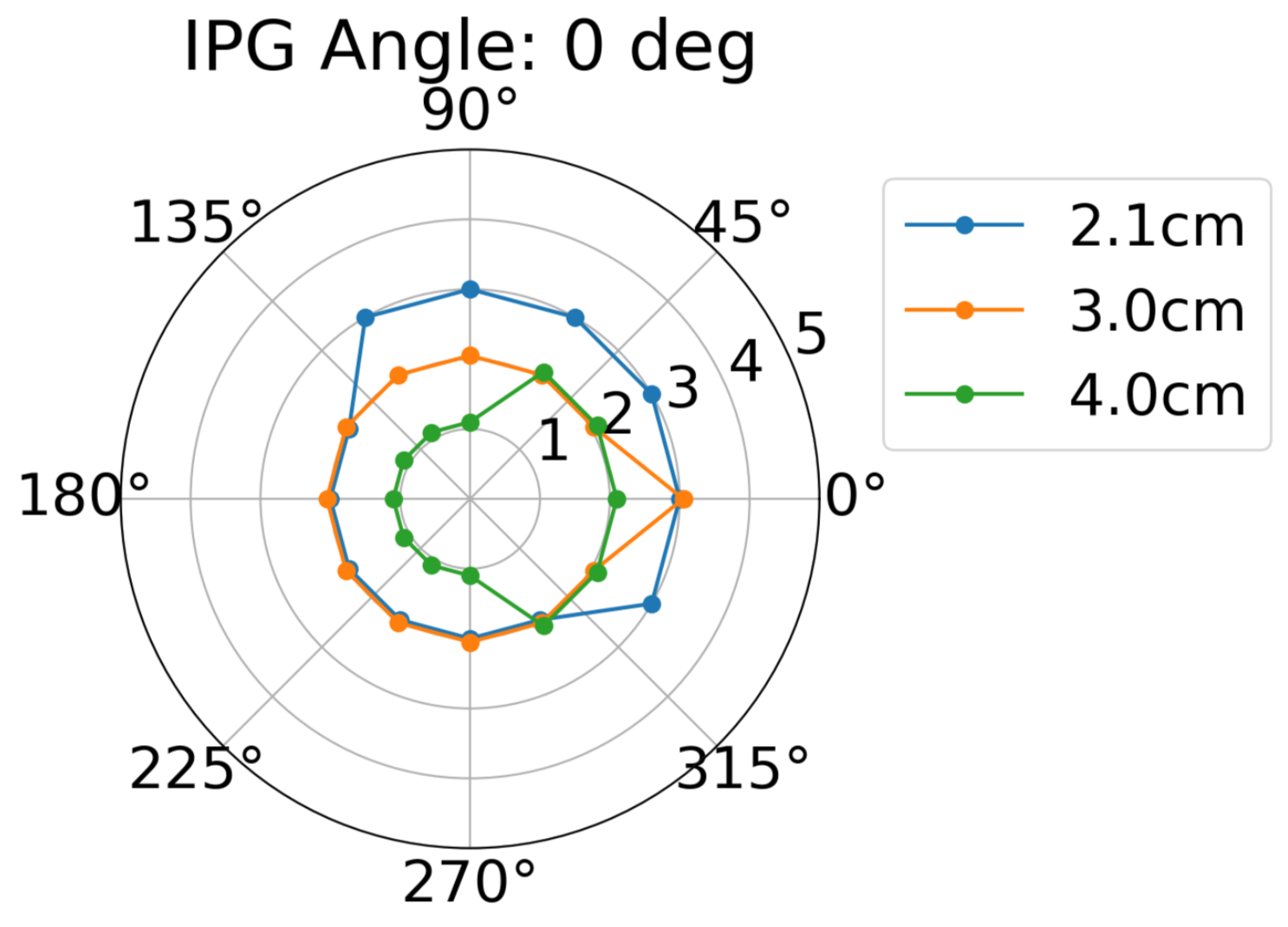


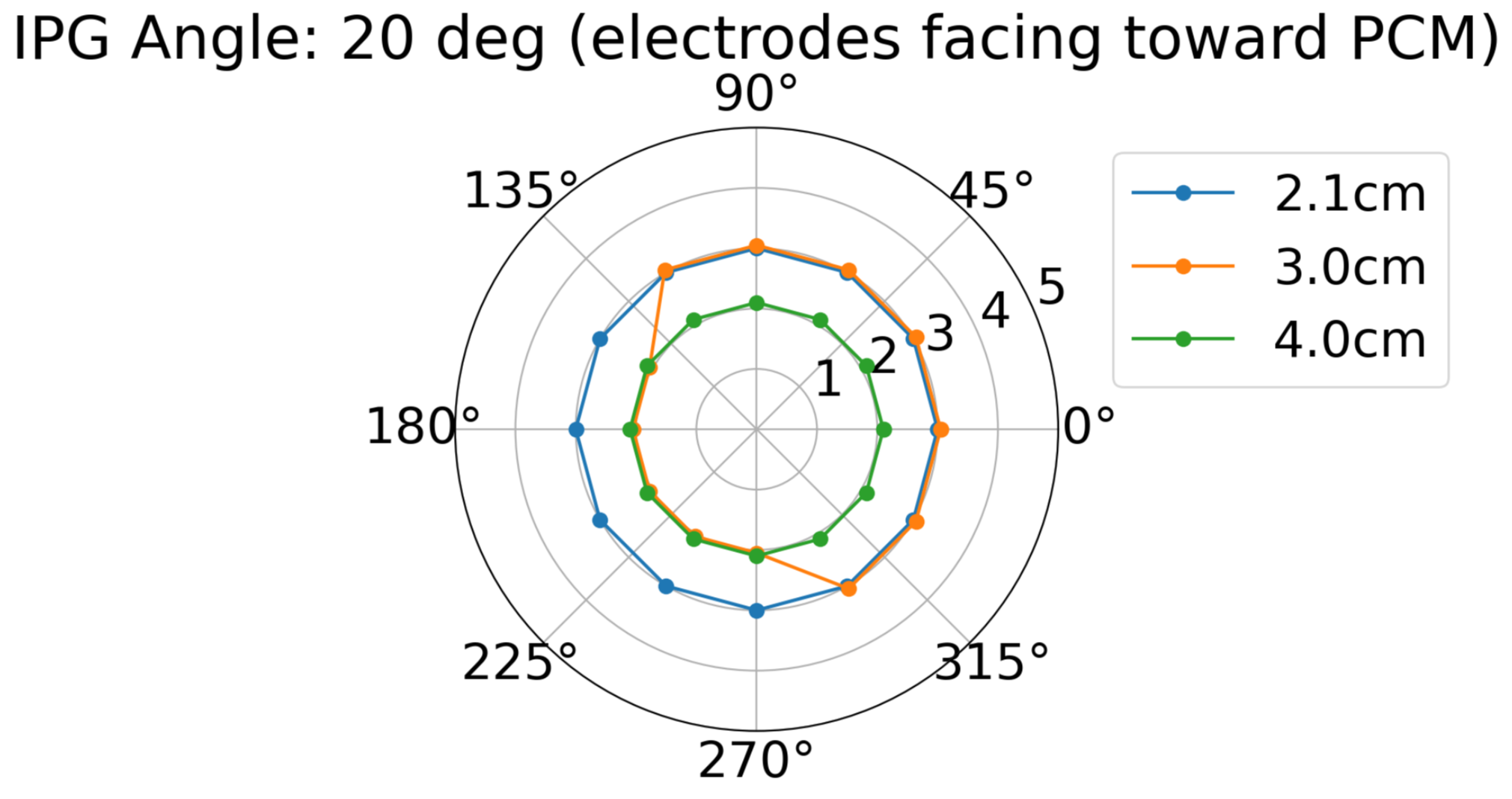

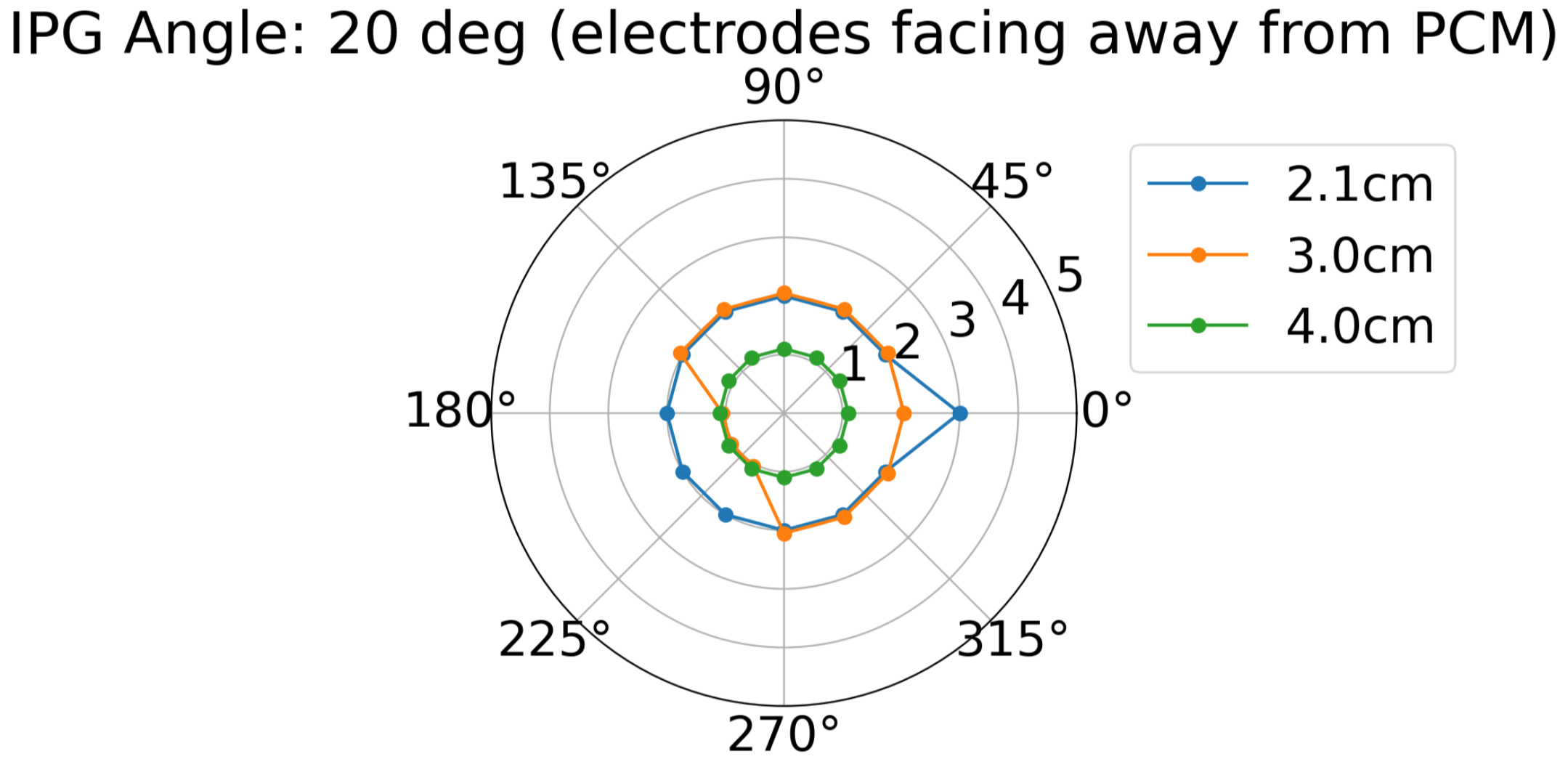


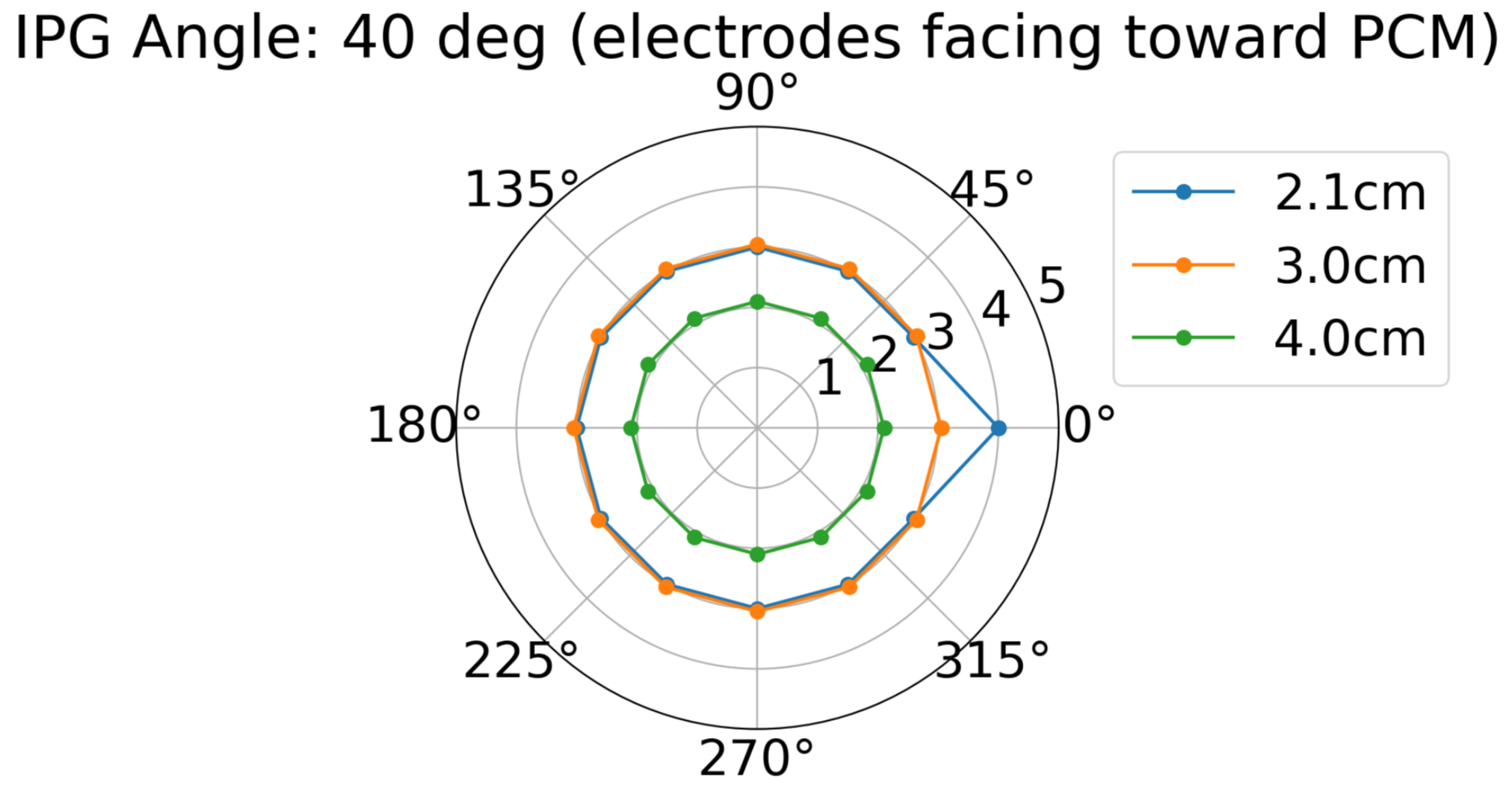

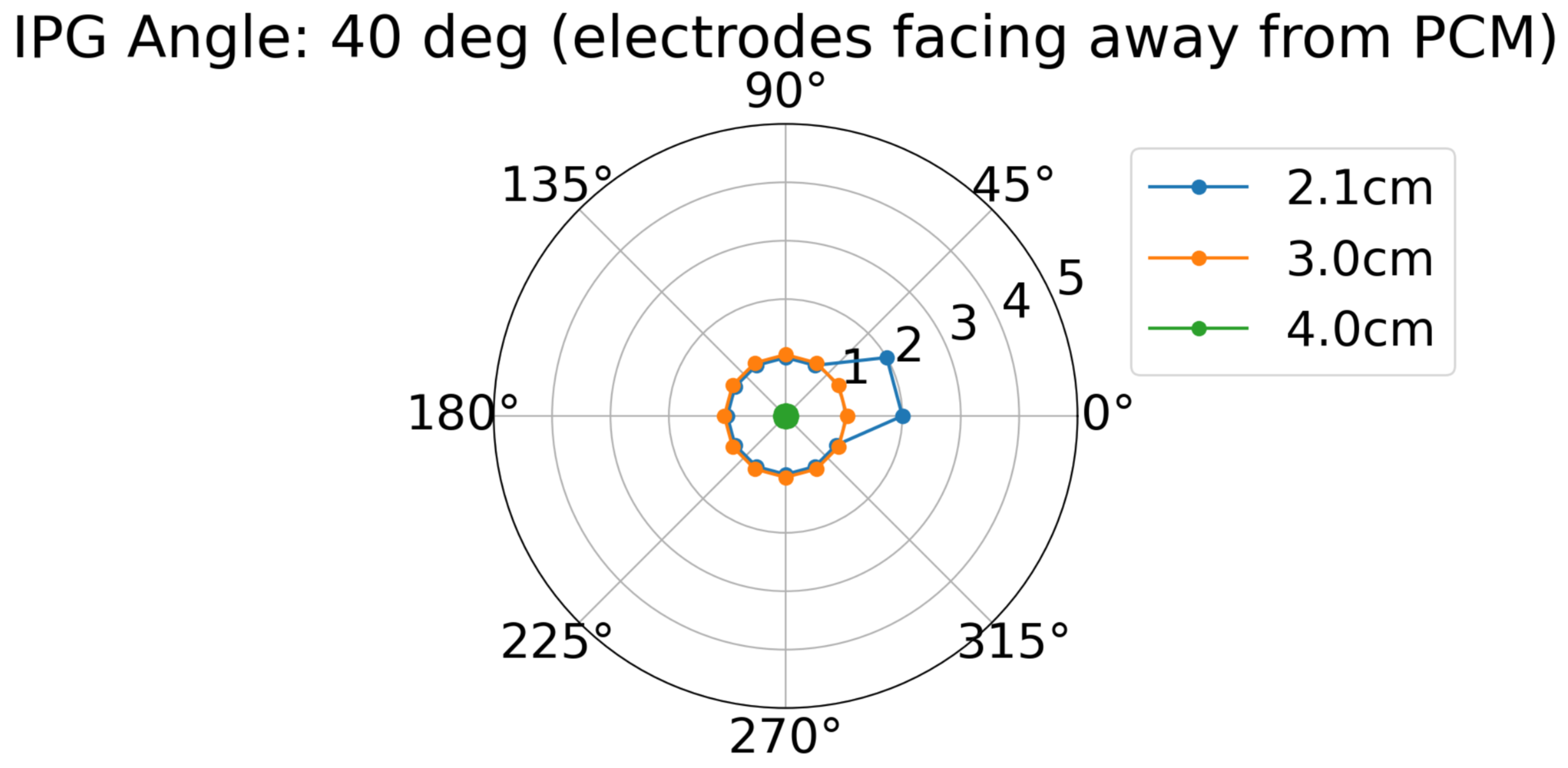


**Supplementary Figure 1: Benchtop testing of MEPS and PCM communication.** The orientation and distance of the MEPS device relative to the PCM were systematically varied to evaluate their impact on communication between the implant and the external components. Testing demonstrated that the device performs reliably within its intended operational range and orientation. Results indicated that misalignment of the implant’s antennae (defined as the electrodes facing either toward or away from the PCM) modulates the effective communication field, either narrowing or expanding it. The center of each plot corresponds to the PCM's center, with concentric circles representing 1 cm increments from the PCM’s center. Dotted lines denote the boundaries of the effective communication field at the specified distance, with the corresponding angle indicated above each plot.


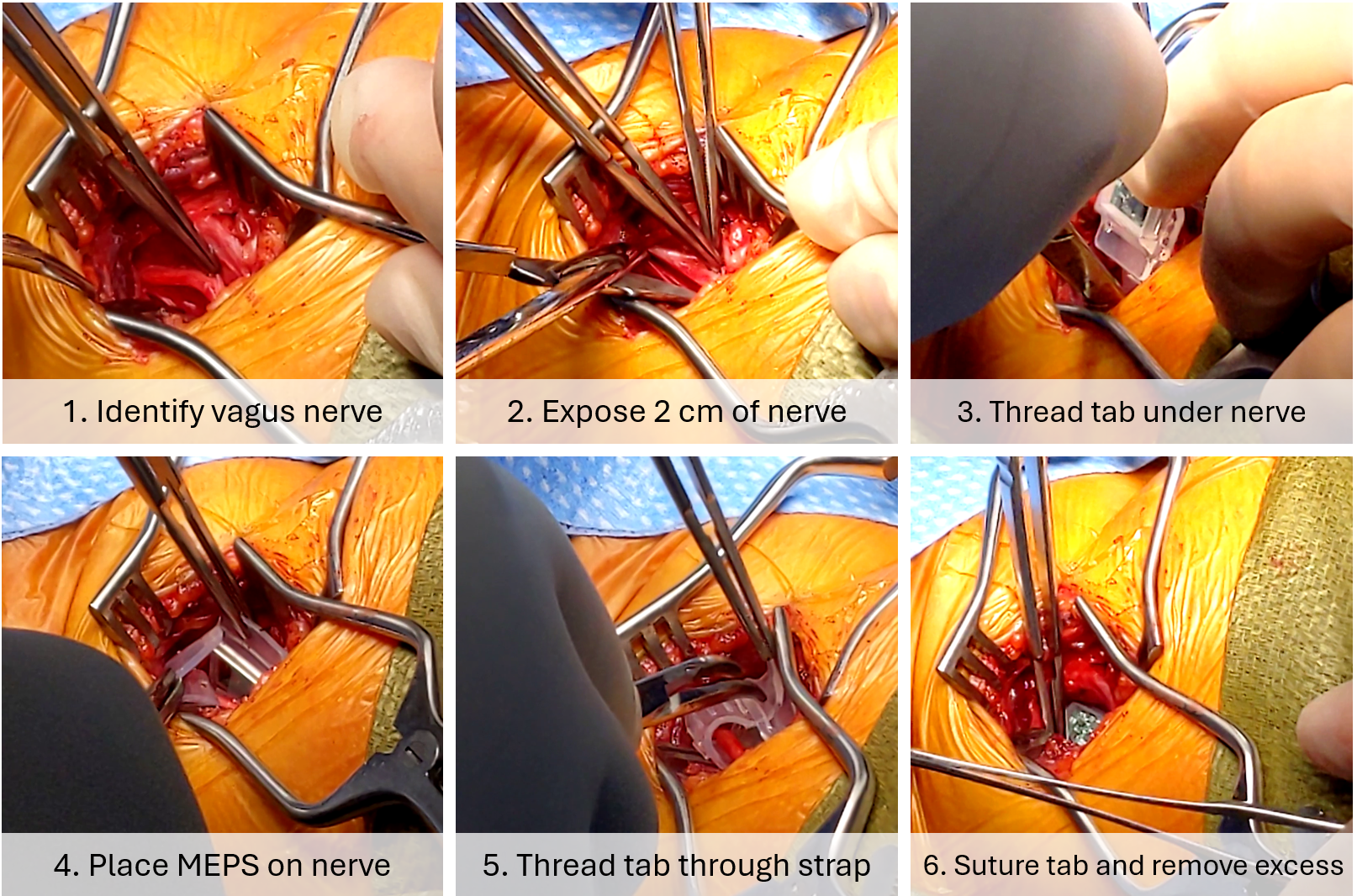


**Supplementary Figure 2: MEPS device implant procedure**. Step-by-step depiction of the MEPS device implantation procedure. (1) The target nerve is identified through surgical exposure. (2) The nerve is isolated for device positioning. (3) The cuff tab is threaded from the medial side around the nerve. (4) The MEPS device, in the cuff, is positioned adjacent to the nerve to expose the silicone loop underneath the cuff. (5) The cuff tab is threaded through the silicone loop and tightened to the appropriate size. (6) The tab is sutured to the cuff body to secure the cuff around the nerve, excess tab material is trimmed, the cuff is rotated to position the MEPS on top of the nerve, and the surgical site is closed.

*Posttraumatic Stress Disorder Study Criteria (NCT04064762)*

Inclusion Criteria

- Patients diagnosed with chronic PTSD for at least 3 months based on the DSM-5 criteria (PSSI-5 score of 9-45)
- Appropriate candidate for VNS implantation
- Adult, aged 18-64
- Provision of signed and dated informed consent form
- Willingness to comply with all study procedures and availability for the duration of the study
- Right vocal cord has normal movement when assessed by laryngoscopy
- Women of reproductive potential must use contraceptive protection

Exclusion Criteria

- PSSI-5 score of 46 or greater (very severe symptoms)
- Current substance dependence
- Currently undergoing prolonged exposure therapy elsewhere
- Concomitant clinically significant brain injuries
- Prior injury to vagus nerve
- Prior or current treatment with vagus nerve stimulation
- Participant receiving any therapy (medication or otherwise) that would interfere with VNS
- Planning to become pregnant, currently pregnant, or lactating
- Clinical complications that hinder or contraindicate the surgical procedure
- Cognitive impairment that would interfere with study participation, confirmed by medical evaluation (will be assessed through chart review and with the Cognistat)
- Psychological conditions such as schizophrenia, bipolar disorder, or psychosis that could interfere with study participation and follow-up
- Participation in other interventional clinical trial
- Participants with known immunodeficiency including participants who are receiving or have received chronic corticosteroids, immunosuppressants, immunostimulating agents or radiation therapy within 6 months
- Significant comorbidities or conditions associated with high risk for surgical or anesthetic survival (e.g. renal failure, unstable cardiac disease, poorly controlled diabetes, immunosuppression, etc.).
- Active neoplastic disease.
- Significant local circulatory problems that would interfere with device implantation and communication, (e.g. thrombophlebitis and lymphedema).
- Any condition which, in the judgment of the Investigator, would preclude adequate evaluation of device's safety and performance.
- Incarceration or legal detention
- Non-English speaking
- Patients who are acutely suicidal and/or have been admitted for a suicide attempt

*Spinal Cord Injury Study Criteria (NCT04288245)*

Inclusion Criteria:

- Provision of signed and dated informed consent form
- Stated willingness to comply with all study procedures and availability for the duration of the study
- Adult, aged 18-64
- In good general health as evidenced by medical history and diagnosed with first time cervical spinal cord injury resulting in an ASIA grade B, C, or D, and level 1 or better motor function as described by the International Standards for Neurological Classification of Spinal Cord Injury (ISNCSCI).
- SCI caused by trauma that occurred ≥ 12 months prior to enrollment
- Meets all clinical criteria for the surgical VNS implantation as determined by the PI, surgeon, and anesthesiologist
- Must demonstrate some residual upper limb and hand movement in either arm
- Appropriate candidate for VNS implantation
- Willing and able to comply with the study protocol

Exclusion Criteria:

- Spinal cord injuries by sharp objects, firearms, and non-traumatic or congenital causes, even if at different levels of the spinal cord
- Any evidence of recurrent laryngeal nerve injury (Evident during required laryngoscopy for all participants with Prior right-sided anterior cervical surgery- done prior to randomization)
- Excessive scar tissue marking implantation unsafe (evident at surgery)
- Concomitant clinically significant brain injuries
- Prior injury to vagus nerve
- Prior or current treatment with vagus nerve stimulation
- Participant receiving any therapy (medication or otherwise) that would interfere with VNS
- Pregnancy or lactation
- Clinical complications that hinder or contraindicate the surgical procedure
- Psychiatric disorders, psychosocial, and/or cognitive impairment that would interfere with study participation, as assessed by medical evaluation
- Abusive use of alcohol and/or illegal substances use
- Participation in other interventional clinical trial
- Participants with known immunodeficiency including participants who are receiving or have received chronic corticosteroids, immunosuppressants, immunostimulating agents or radiation therapy within 6 months
- Significant comorbidities or conditions associated with high risk for surgical or anesthetic survival (e.g. renal failure, peripheral vascular disease, unstable cardiac disease, poorly controlled diabetes, immunosuppression, etc.).
- Active neoplastic disease.
- Participants with significant local circulatory problems, (e.g. thrombophlebitis and lymphedema).
- Participants with any medical condition or other circumstances that might interfere with their ability to return for follow-up visits in the judgment of the Investigator, including systemic illness, neuromuscular, neurosensory, or musculoskeletal deficiency that would render the participant unable to perform appropriate postoperative rehabilitation.
- Any condition which, in the judgment of the Investigator, would preclude adequate evaluation of device's safety and performance.
- Aphasia and other cognitive deficits that make understanding the potential risks and benefits of the study impossible for participant. Inability to personally provide informed consent.
- A recent history of syncope
- A recent history of dysphagia
- Currently require, or are likely to require diathermy
- Significant respiratory issues that would interfere with participation
- Non-English speaking
- Patients who are acutely suicidal and/or have been admitted for a suicide attempt
- Incarceration or legal detention

*Stroke Study Criteria (NCT04534556)*

Inclusion Criteria:

- Provision of signed and dated informed consent form
- Stated willingness to comply with all study procedures and availability for the duration of the study
- Adult, aged 22-79
- Ischemic or hemorrhagic stroke that occurred ≥ 12 months prior to enrollment
- UEFM score of 20 to 50
- Modified Rankin Score of 2, 3, or 4
- Right vocal cord has normal movement when assessed by laryngoscopy
- Women of reproductive potential must use contraceptive protection
- Meets all clinical criteria for the surgical VNS implantation as determined by the PI, surgeon, and anesthesiologist

Exclusion Criteria

- Deficits in language or attention that interfere with study participation
- Severe spasticity (Modified Ashworth ≥ 3)
- Medical or mental instability that would likely interfere with study protocol
- Receiving any therapy (medication or otherwise) that would interfere with VNS, such as drugs that perturb neurotransmitter action (anticholinergics, adrenergic blockers, etc.)
- Presence of any other implanted electrical stimulation device
- Prior injury to vagus nerve
- Lactating, pregnant, or plan to become pregnant
- Participation in another interventional clinical trial
- Clinical complications that hinder or contraindicate the surgical procedure
- Abusive use of alcohol and/or illegal substances use
- Participants with sickle cell, lupus, clotting disorders or active neoplastic disease.
- Participants with any any medical condition or other circumstances that might interfere with their ability to return for follow-up visits in the judgment of the Investigator.
- Any condition which, in the judgment of the Investigator, would preclude adequate evaluation of device's safety and performance.
- Recent history of syncope
- Recent history of dysphagia
- Current or anticipated requirement for diathermy
- Uncontrolled hypertension
- Diagnosed with Cerebral amyloid angiopathy
